# SELDI-TOF mass spectrometry of High-Density Lipoprotein

**DOI:** 10.1186/1477-5956-5-15

**Published:** 2007-09-06

**Authors:** Johannes HM Levels, Boris Bleijlevens, Farhad Rezaee, Johannes MFG Aerts, Joost CM Meijers

**Affiliations:** 1 Department of Experimental Vascular Medicine, Academic Medical Center, University of Amsterdam, Meibergdreef 9, 1105 AZ Amsterdam, The Netherlands; 2 Clinical Proteomics Facility, Department of Medical Biochemistry, Academic Medical Center, University of Amsterdam, Meibergdreef 9, 1105 AZ Amsterdam, The Netherlands; 3 Division of Molecular Biosciences, Faculty of Natural Sciences, Imperial College London, South Kensington Campus, London SW7 2AZ, UK; 4 Center for Medical Biomics, Medical University of Groningen, Anthonius Deusinglaan 1, 9713 AV Groningen, The Netherlands; 5 Department of Vascular Medicine, Academic Medical Center, University of Amsterdam, Meibergdreef 9, 1105 AZ Amsterdam, The Netherlands

## Abstract

**Background:**

High-Density Lipoprotein (HDL), one of the main plasma lipoproteins, serves as a docking station for proteins involved in inflammation, coagulation, and lipid metabolism.

**Methods:**

To elucidate the protein composition of HDL, we employed SELDI-TOF mass spectrometry as a potential high-throughput proteomic candidate for protein profiling of HDL. HDL derived from normolipemic individuals was captured on PS20 protein-chips using covalently bound antibodies against apo A-I or A-II.

**Results:**

After optimisation, on-chip capture of HDL particles directly from plasma or from pre-purified HDL resulted in comparable fingerprints confirming specific capture of HDL. Depending on the capture antibody some differences in the fingerprint were observed. The most detailed fingerprint was observed up to 50 kDa; approximately 95 peaks were detected in the 3–50 kDa molecular mass range. Between 50 and 160 kDa, 27 more peaks were detected.

**Conclusion:**

Based on these results, SELDI-TOF MS may be a suitable high-throughput candidate for HDL protein profiling and marker search. This approach may be used to *i) *investigate the underlying mechanisms that lead to increased atherothrombotic risk and *ii) *to investigate the atherothrombotic state of an individual.

## Background

Vascular disease, and particularly atherothrombotic disease, is one of the major causes of death worldwide. Dyslipidemia, one of the primary risk factors of atherosclerosis, is characterized by elevated levels of atherogenic lipoproteins (chylomicron remnants, very low-density lipoprotein (VLDL) and low-density lipoprotein (LDL)). In contrast, high-density lipoprotein (HDL) is inversely associated with the risk for cardiovascular disease [1].

Intensive research over the past decade has elucidated the involvement of multiple pathways in atherogenesis, such as a long-term inflammatory and immune responses [2,3], hyper- and hypolipidemia, oxidation of LDL and HDL, coagulation and fibrinolysis [4-6]. Key markers of inflammation and the innate immune response include C-reactive protein (CRP), interleukin-6 (IL-6), tumor necrosis factor-alpha, and several adhesion molecules [7]. Apart from decreases in the lipid content of HDL upon the inflammatory reaction [8-10], also the HDL protein composition markedly changes during the course of acute infection and consequent systemic inflammatory response [11-14]. These modified HDL particles are known as "acute phase HDL" [2,15-18]. According to the Ross hypothesis also hemostatic abnormalities contribute to the hypercoagulable state [19] that may result in a pro-atherogenic risk profile.

The balance between the HDL protein composition and the different functions of HDL such as stimulation of reverse cholesterol transport, antioxidant effects, anti-inflammatory effects, attenuation of endothelial dysfunction, reduction of lipoprotein retention and the antithrombotic and profibrinolytic effects may have an impact on the progress of the atherothrombotic disease.

A proteomic approach covering a broad range of protein analysis has shown to be a good candidate to address the HDL protein composition [20-22]. In particular, surface-enhanced laser desorption ionization time-of flight mass spectrometry (SELDI-TOF MS) employs a high-throughput, sensitive and reproducible method for the analysis of complex protein mixtures [23,24]. Protein fingerprints can be achieved after retaining a selection of a complex protein mixture array by chemical, physical or functional properties [25,26]. For LDL protein analysis this has already been established to be a suitable approach [27] which is for HDL lacking to date.

In this study, we investigated SELDI-TOF MS analysis of on-chip immuno-captured native HDL particles using antibodies against apo A-I or apo A-II. We propose that this method is suitable for screening of HDL or its HDL sub-populations in the search for biomarkers related to cardiovascular disease.

## Results

In this study SELDI-TOF mass spectrometry was used to determine protein profiles of HDL associated proteins directly from plasma. To prevent disturbance of non-HDL components, an immunocapture procedure was performed using anti-apo A-I or apo-A-II capture antibodies. Immuno-captured HDL contained no contaminants when compared to a HDL gel filtration lipid profile of normal plasma. Especially, no apo B mass could be detected in immuno-purified HDL in which the same capture antibodies were used as [28]. As previously shown, 2-D gel profiles of HDL isolated with the same anti-apo A-I antibodies revealed a number of proteins that appeared to be associated with HDL [20,21]. Similar results were obtained using anti-apo A-II as capturing antibody. These data demonstrated the feasibility of HDL-immunocapturing from plasma.

### Anti apo A-I and A-II immuno-capture characteristics on a SELDI protein chip

**Figure 1:**
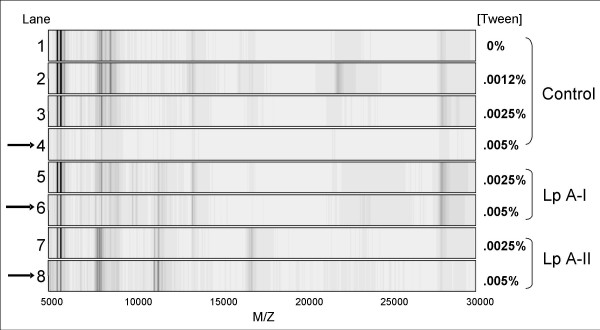
**Virtual gel-view fingerprints of binding of HDL to a PS20 protein-chip**. Lanes 1–4; Non-specific protein binding in the absence of specific antibodies **(Control) **in the presence of indicated Tween-20 concentrations in the final wash step (See materials and methods). Lanes 5–6; HDL capture with anti-apo A-I **(Lp A-I)**, Lanes 7–8; HDL capture with anti-apo A-II **(Lp A-II)**. The arrows indicate the conditions used in all further experiments.

The immunocapture procedure was executed on a SELDI protein chip to allow analysis of HDL bound proteins by mass spectrometry. Using antibodies against apo A-I or apo A-II a number of proteins could be visualized by gel view (Fig. 1). The wash stringency specifically determined the outcome of the fingerprint (Fig 1, lane 1–4). In the absence of antibodies on the SELDI chip, non-specific binding of plasma components was observed. Depending on the presence of Tween-20 in the final wash step this non-specific binding was virtually abolished (Fig. 1, lane 4). In the presence of antibodies (Fig. 1, lane 6–8) specific HDL associated proteins were detected even in the presence of 0.005 % (v/v) Tween-20. More stringent wash conditions i.e. high salt or high Tween concentrations resulted in a complete abolishment of non-specific components but also in a reduction of specific markers such as apo A-I and apo A-II in case of anti A-II or A-I capture respectively. (not shown). The presence of the 0.005 % (v/v) Tween-20 in the last wash step appeared to virtually eliminate non-specific IgG and albumin contamination without loss of antibody-mediated capture, and was therefore used in further experiments.

The Intra CVs of the HDL capture was determined using a plasma pool of more than 200 people. This plasma-pool had been aliquoted, snap frozen on liquid nitrogen and stored at -80°C. Intra CVs ranged from 10 to 30 % over the 3–150 kDa m/z range. The Inter CV range was also determined in both captures, in the 3–50 kDa range the inter CVs ranged from 10 to 25 % and in the 50–160 kDa m/z from 20 to 35 %.

The individual variation (normal variation) was determined in 20 subjects having normal HDL levels with respect to apo A-I and cholesterol content. The inter-individual CVs were 10 to 35 % in the 3 kDa-10 kDa m/z range and 15 to 40 % in the 10–150 kDa m/z range.

To investigate the dynamic range of the capture a dilution series of plasma with apo A-I levels ranging from 1.78 umolar to 23 pmolar were assessed. A decrease of the apo A-I signal was seen in the range from 14 nmolar to 110 pmolar. The HDL specific pattern virtually did not change except for the intensity of the peaks. Due to the fully focussed laser energy to the on-chip coupled IgG, the IgG specific pattern appeared to be enhanced upon the decrease of the concentration of HDL. However, in the setup of the experiments, the chip was saturated with HDL particles. Similar results were also seen for the anti apo A-II capture (data not shown).

### Specificity of the HDL capture

**Figure 2:**
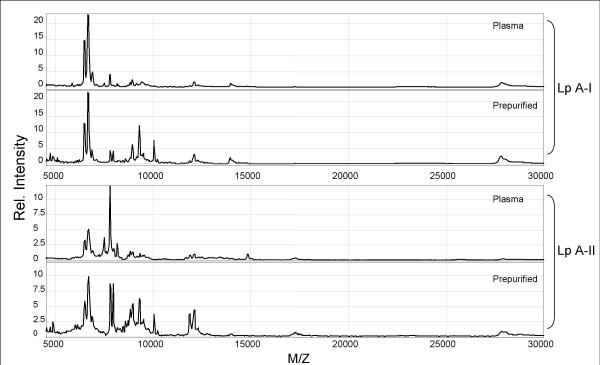
**Representative examples of the fingerprint of plasma HDL and pre-purified HDL after capture on a PS-20 protein-chip**. Pre-purified HDL (by gel filtration) and plasma HDL of the same individual was subjected to SELDI-TOF analysis according to the conditions as described in the materials and methods section. Shown are the fingerprints obtained after capture with the indicated monoclonal antibodies against apo A-I (Lp A-I) or apo A-II (Lp A-II).

The protein composition of immuno-captured HDL from plasma was compared with that of HDL isolated from plasma by gel filtration (Fig. 2). Both approaches resulted in comparable fingerprints, indicating that immunocapturing of HDL is a good alternative for purification and a much easier method for scale up.

**Figure 3:**
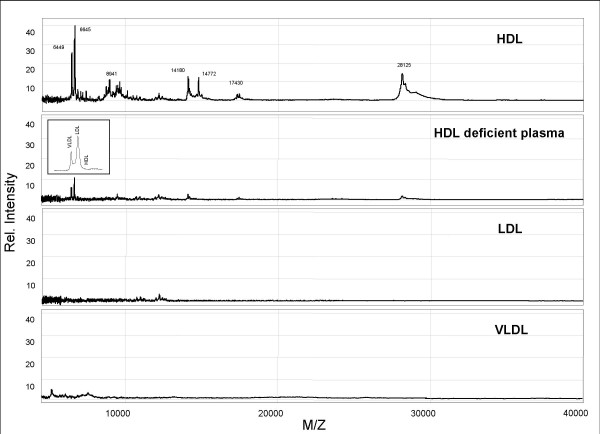
**Fingerprints HDL, HDL deficient plasma, LDL and VLDL**. All particles were first isolated by gel filtration were subjected to SELDI-TOF analysis according to the methods as described in the M&M section. **Upper and Second panel: **A spectrum of HDL from a normal subject and HDL deficient plasma (Tangier patient having virtually no HDL cholesterol [29]). **Insert**: The cholesterol gel filtration profile of HDL deficient plasma showing only the presence of VLDL and LDL and no lipid containing HDL. **Third and lower panel: **LDL and VLDL spectra showing virtually no HDL specific pattern.

Next, the specificity of the HDL capture was investigated by exposing other plasma components to the antibodies on the immuno-capture chip. Hardly any signal was observed when LDL or VLDL was exposed to the SELDI-chip (Fig. 3). Moreover, exposure of plasma of a Tangier patient, with known low HDL cholesterol levels [29], still resulted in HDL specific protein markers although in a very low abundancy.

### Apo A-II isoforms

**Figure 4:**
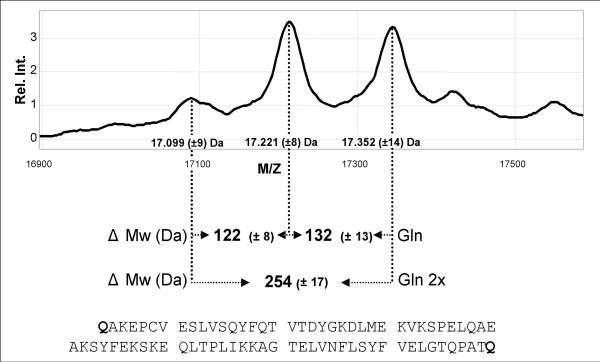
**Analysis of Apo A-II isoforms in a normal individual**. The M/Z mass ratios of the three isoforms of apo A-II are indicated in the peaks after HDL capture by anti-apo A-II antibodies (see material and methods). The mean mass differences between the peaks are indicated as ΔMw. The amino acid sequence of apo A-II is indicated at the bottom in which the "end"-terminal glutamine residues have been indicated in bold.

Apo A-II was visible as a dimer by mass spectrometry. Upon further inspection, the apo A-II species consisted of three major isoforms at 17099, 17221 and 17352 M/Z, respectively (Fig. 4). These isoforms were observed due to the absence of one or two "end"- terminal glutamine residue per dimer. Each loss of one Gln residue resulted in a M/Z decrease of 128 Da and in case of a loss of 2 Gln residues a decrease of 256 Da was observed (Fig. 4). These observed M/Z ratios appeared to be in agreement with the theoretic calculated M/Z's of the three apo A-II isoforms.

### Differences between HDL fingerprints after apo A-I or apo A-II capture

**Figure 5:**
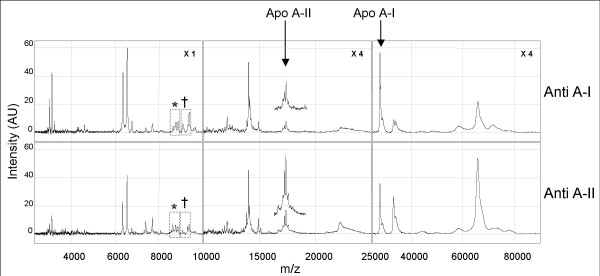
**Fingerprints of plasma HDL captured on a PS20 protein-chip**. Shown are the HDL fingerprints captured from plasma with monoclonal antibodies against apo A-I (Upper panel) or apo A-II (Lower panel). A molecular range up to 80 kDa is presented using different magnifications of the intensities as indicated for the three mass ranges. The apo A-I protein peak is observed at 28 kDa. **Insert**: An extra 2.5 magnification of native apolipoprotein A-II (17 kDa) as detected by SELDI-TOF MS.* corresponds with m/z of monomeric apo A-II. † corresponds with the apo C III.

In figure 5, a typical example of a HDL fingerprint is presented. The apo A-I (28 kDa) and the dimeric form of apo A-II (17.4 kDa) clearly recovered in our experimental setup at the indicated masses. Independent of the use of the antibodies, both apo A-I and apo A-II were observed. These findings confirm the published observations with other MS approaches than SELDI-TOF MS [30,31]. When using the anti A-II capture a less intense apo A-I and a more intense apo A-II peak could be observed whereas in the anti A-II capture the pattern was the reverse. Furthermore, as indicated in the highlighted areas, (Fig. 5) also differences in the obtained fingerprints could be observed, which were HDL capture approach dependent.

**Figure 6:**
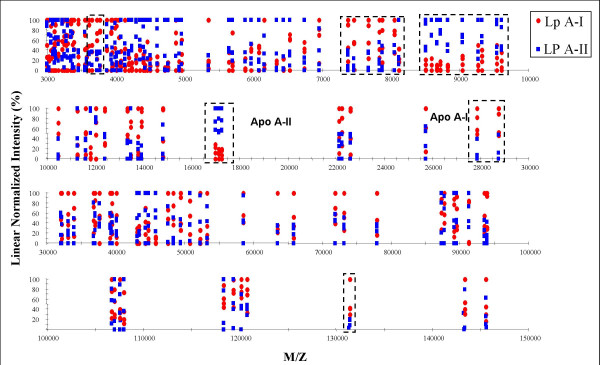
**Normalized plot of the spectra of two HDL capture types**. Spectra were obtained after capture of HDL from plasma of 4 healthy individuals. The highlighted areas show capture-type dependent differences between the apo A-I (Lp A-I) and apo A-II (Lp A-II) (p < 0.05).

The most detailed fingerprint was observed up to 50 kDa; approximately 95 peaks were detected in the 3–50 kDa molecular mass range. Between 50 and 160 kDa, 27 more peaks were detected. A linear normalized plot (Fig. 6) of the complete spectrum demonstrates an overview of all detected peaks of both capture types. Notably at least in 5 regions of the spectrum, capture dependent differences (approximately 33% of the total spectrum) could be observed. This indicates that the used approach identified two similar but distinct HDL pools depending on the capture antibody.

## Discussion

It has been recognised that HDL is a carrier of a large number of proteins with variable binding affinities [21]. In this study, a proteomic approach was used exploiting the potential of SELDI TOF MS as a high-throughput screening tool for HDL profiling. Immuno-capturing of HDL in combination with subsequent mass-spectrometry analysis has been validated as a tool for HDL fingerprinting. Capturing of HDL on the protein chips was found to be reproducible. Depending on the capture (anti-apo A-I or A-II) antibody, differences in HDL subpopulations were revealed in the SELDI-TOF ms fingerprints.

The wash stringency between the different incubation steps highly influenced the outcome of the fingerprint. Here, the selected wash stringency was optimized by the use of Tween-20 in the final wash step to reduce non-specific binding. This resulted in minor albumin contamination. Furthermore, only the capture antibody IgG, coupled onto the SELDI protein-chip, and not plasma IgG could be detected even in the case when very high laser intensities were used (>220 relative units). It can be concluded that this setup is a fast purification alternative of HDL particles prior to the protein profiling.

Specificity of the capturing procedure was demonstrated in 5 ways: 1. The used antibodies captured apo A-I and apo A-II containing particles as demonstrated in Figures 2 and 3. The 28 kDa peak represents apo A-I and the 17 kDa represents the dimeric form of apo A-II [30,32]; 2. Capturing with anti-apo A-I or A-II antibodies resulted in comparable fingerprints; 3. HDL pre-purified by gel filtration or captured from plasma revealed comparable fingerprints. Only a variation in intensity of the peaks was observed which was probably due to different levels of the total particle load on the SELDI-chip; 4. LDL exposed to the protein chips showed no "HDL" specific profile; 5. the highly abundant proteins in plasma (albumin and IgG) could not or hardly be detected in the fingerprints whereas the most detailed fingerprint was observed in the low mass range of the spectrum. Analysis of HDL previously confirmed the presence of a complex range of small peptides in the mass range from 1000 to 5000 M/Z [33].

These lines of evidence suggested that the capturing of HDL from plasma on a SELDI chip with antibodies is a robust and specific procedure without appreciable contamination from plasma components and other lipoproteins.

Additional evidence confirming that the integrity of the HDL particle was maintained during the capture procedure, was the presence of dimeric apo A-II (Fig. 4). This is in concordance with previous findings using other MS approaches [30,34]. From these observations it can be concluded that the obtained profiles were an outcome of captured native HDL particles onto the SELDI chip. Our proteomic profiling demonstrated that the dimeric form of apo A-II consisted of three species (Fig. 4). These isoforms were observed due to the absence of probably one or two C- terminal glutamine residue per dimer and is in concordance with findings of others who have used a different proteomic approach [30]. This clearly proved the feasibility of SELDI-TOF MS to discriminate between apo A-II isoforms on amino acid level. We therefore propose that the obtained resolution by this profile approach also might be useful for the identification of isoforms in other HDL associated proteins.

Depending on the use of anti-apo A-I or apo A-II antibodies, slight differences between the profiles could be observed (Fig 6). Specifically in the most sensitive measurable mass range up to 80.000 M/Z SELDI TOF MS could be used for discrimination of HDL subtypes and could be a useful extension to the currently available analysis tools for protein composition of HDL.

The most striking aspect of the protein chip approach is that the preparation of the HDL consumes a minimum of time compared to the classic preparation techniques for lipid preparation e.g. ultracentrifugation, native gel electrophoresis or gel filtration. Moreover, in order to obtain a detailed fingerprint of the HDL associated proteins SELDI-TOF MS is much less labour intensive and much lower in sample consumption compared to other proteomic approaches. We are aware that there are some limitations for this approach. SELDI-TOF ms as such is not a approach for direct protein identification, but as marker search this approach has high potential. Moreover, due to the defined wash stringency one should keep in mind that an under- or over- estimation of the total HDL proteome may occur. Particularly in case of higher dilution of plasma samples, markers of minor abundant HDL subfractions might be lost. This proposed approach might despite the selective "plasma proteomics" be of more value compared to complete plasma proteomics. Now, work is in progress to investigate whether HDL protein fingerprinting could be a suitable tool in early diagnosis of patients at high atherothrombotic risk followed by steps towards protein identification.

We conclude that SELDI-TOF mass-spectrometry offers a high throughput approach to study HDL composition. Our goal for future studies is to determine whether changes in HDL protein composition contribute to atherothrombotic events. In addition, identification of HDL protein components could also lead to the discovery of new biomarkers for the diagnosis and early detection of atherosclerotic diseases and may contribute to the development of more effective therapeutic strategies.

## Methods

### Sample collection

For lipoprotein measurements, blood was collected in non-additive or EDTA (10 mM final) containing vacutainer tubes (Becton Dickinson, Mountain View, CA) from healthy volunteers. For the determination of the inter and Intra CV of the HDL capture a plasma pool of more than 200 people was used. The individual variation (normal variation) was determined in 20 subjects having normal HDL levels with respect to apo A-I and cholesterol content. Plasma was prepared and stored at -80°C until batch-wise assessment. Monoclonal antibodies (anti apo A-I and A-II), specific for the capture of native HDL-particles containing apo A-I or apo A-II [35,36], were obtained from Acris GmbH (Hiddenhausen, Germany) and from dr. J Fruchard, University of Lille, France. As determined by surface plasmon resonance, dissociation of HDL from the antibodies against apo A-I and apo A-II appeared to be negligible (K_*d *_< 10^-5 ^s^-1^).

### Preparative isolation of lipoproteins by Fast Performance Liquid Chromatography

Lipoprotein fractions were isolated from 100 μl plasma samples by size-exclusion chromatography using a Superose 6 HR 10/30 (Pharmacia Biotech, Uppsala, Sweden) column at a flow rate of 0.3 ml/min with inline fluorescence and UV detection [37]. VLDL, LDL and HDL containing fractions were collected and concentrated with Centricon-100 (Beverley, MA, USA) concentrator filters to a final volume of 100 μl. Samples were used immediately or frozen in liquid nitrogen and stored at -80°C.

### Cholesterol analysis (FPLC)

Cholesterol concentrations in the main lipoprotein classes (VLDL, LDL and HDL) were determined using high performance gel filtration chromatography (HPGC) as described elsewhere [38]. Total cholesterol was determined by PAP 250 cholesterol enzymatic methods (Biomerieux, Le Fontanille, France). Commercially available lipid plasma standards (low, medium and high) were used for total cholesterol quantitative analysis (SKZL, Nijmegen, the Netherlands) of FPLC separated lipoproteins.

### Sample preparation and SELDI-TOF MS analysis

#### Coating of antibodies

A 5 μL mixture containing 2.8 nM anti-apo A-I or A-II monoclonal antibodies, 3 μM ethylenediamine and 0.1 M Na_2_SO_4 _was added per spot of a PS-20 protein chip (Ciphergen Biosystems, Fremont, CA, USA) and covalent binding of antibodies through primary amine-epoxide chemistry was achieved by incubating the chip in a humid chamber overnight at 4°C. Excess antibody was removed by 1 wash with distilled water and subsequently free amine-binding places were blocked by incubating the chip for 30 min at room temperature with 1 M Tris buffer (pH 8.0).

#### HDL capture

After mounting the PS-20 protein chip(s) in a 96 wells bioprocessor, 100 μL diluted plasma aliquots (1:2 diluted with Tris Buffered Saline pH 7.4) or purified HDL were applied onto single SELDI spots and were allowed to bind for 2 hours at room temperature on a horizontal shaker. The protein chips were washed 4 times with TBS for 10 minutes, followed by a 5 minutes TBS-Tween (0.005%) rinse unless indicated otherwise. A final wash step with Hepes solution (5 mM) was carried out to remove the excess of salt. All spots were allowed to dry and subsequently 1.2 μL sinapinic acid (10 mg/ml) in a 50/49.9/0.1 % acetonitril/H_2_O/trifluoric-acid mix was applied on each spot. All chips were air dried and stored at room temperature in the dark. The binding capacity of the coated chips was approximately 5–10% of the total HDL pool present in plasma of normal individuals resulting in the chip surface to be the limiting step in the capture procedure.

#### SELDI-TOF analysis

Analysis was carried out using a PBS IIc protein chip reader (Ciphergen Biosystems) using an automated data collection protocol within the Protein-Chip Software (version 3.1). Data were collected up to 200 kDa. Laser intensity was set in a range from 190 to 220 relative units and the focus mass was set to either 17 kDa in case of anti A-II or 28 kDa in case of anti A-I capture and in some cases an automatic center focus mass was used. Measurement of the spectra was performed with approximately 100 shots at 13 positions per SELDI spot. Calibration was done using a protein calibration chip (Ciphergen). Spectra were normalised on total ion current. Detected peaks having a signal/noise ratio > 5 were recognized as significant peaks. Differences between capture by apo A-I and apo A-II antibodies was assessed by student's t-test.

## Abbreviations

MALDI-TOF-MS, Matrix Assisted Laser Desorption Ionization-Time of Flight-Mass Spectrometry; *m*/*z *value, mass-to-charge ratio; PBS IIc, Protein Biosystem IIc

## Competing interests

The author(s) declare that they have no competing interests.

## Authors' contributions

Authors 1, 2 and 3 JHML, BB and FR designed and carried out the SELDI-TOF and additional experiments. Authors 4 and 5 JMFGA and JCMM were responsible for the design of the study. All authors were involved in writing the manuscript, and have read and approved the final manuscript.
